# Ethical climate as a moderator between organizational trust and whistle-blowing among nurses and secretaries

**DOI:** 10.12669/pjms.342.14669

**Published:** 2018

**Authors:** Seda Aydan, Sidika Kaya

**Affiliations:** 1 Dr. Seda Aydan Research Assistant, Faculty of Economics and Administrative Sciences, Department of Health Care Management, Hacettepe University, Ankara, Turkey; 2 Prof. Sidika Kaya Faculty member, Faculty of Economics and Administrative Sciences, Department of Health Care Management, Hacettepe University, Ankara, Turkey

**Keywords:** Ethical climate, Organizational trust, Path analysis, Whistleblowing, Nurses, secretaries

## Abstract

**Objectives:**

To reveal the effect of perception of ethical climate by nurses and secretaries and their level of organizational trust on their whistleblowing intention.

**Methods:**

Nurses and secretaries working in a University Hospital in Ankara, Turkey, were enrolled in the study conducted in 2016. Responses were received from 369 nurses and secretaries working at Clinics and Polyclinics. Path analysis, investigation of structural equation models used while multi-regression analysis was also applied.

**Results:**

According to the regression model, ethical climate dimensions, profession, gender, and work place had significant impact on the whistleblowing intention. According to Path analysis, ethical climate had direct impact of 69% on whistleblowing intention. It was seen that organizational trust had an indirect impact of 27% on the whistleblowing score when ethical climate had a moderator role.

**Conclusion:**

In order to promote whistleblowing in organizations, it is important to keep the ethical climate perception of employees and the level of their organizational trust at high levels.

## INTRODUCTION

Whistleblowing is becoming more important as negative events or behaviours that are subject to whistleblowing in the health sector which causes more concrete and serious consequences that can affect human life. Whistleblowing is defined as “*the act by an employee of informing the public or higher management of unethical or illegal behaviour by an employer or supervisor*” for organizations.^1^

Whistleblowing in the health sector is a controversial issue and there are some problems in reporting mistakes for health care workers.^2^ Health workers are faced with a moral dilemma in trying to solve the negative situation in some cases. Many health workers, who are afraid of retiring to a lower position or being fired from work as a result of whistleblowing, are reluctant to speak about managers.^3^ Gallagher (2010)^4^ has argued that fear of being seen as unfaithful to organization or colleagues, personal retaliation and disturbing patient privacy in case of reporting outside the organization may lead to incomplete reporting. It is stated that employees who prefer whistleblowing is closely followed within the organization, taken on the black list, and exposed to criticism by colleagues.^5^

Researchers have identified the need for whistleblowing as a key factor in highlighting the need for health care reform and maintaining health care safety and quality.^2^ This issue should be emphasized due to the fact that the whistleblowing has important effects and has feature of preventing the negativities and improving the services in the health sector.

Situations that may be subject to whistleblowing in health institutions may be financial and administrative misconduct, violation of law and ethical values, or medical malpractices. In the health sector, malpractices or unethical behaviours could give rise to important outcomes having impact on human lives. It is important that health professionals work in a cultural environment that encourages them to express their concerns about well-being of patients or other employees. Whistleblowing is one of the main factors in maintaining quality of healthcare and safety. For this reason, it is important to encourage whistleblowing and to determine the factors affecting the whistleblowing.

Ethical climate and organizational trust are thought to be important factors in promoting whistleblowing. The mechanism of reporting negative situations and behaviours is an important basis for the management to know the unethical situations and behaviours and to take corrective steps. The positive feelings of trust that the employees have developed against the employer may also help the whistleblower in whistleblowing process and the problem may be resolved within the organization without moving to the non-organizational institutions.

There are various studies in literature that relate ethical climate perception and organizational trust to some organizational concepts such as job satisfaction, organizational commitment, organizational citizenship, business performance, organizational justice, intention to leave work, team work and mobbing. The studies that reveal the effect of ethical climate and organizational trust on the intention of whistleblowing are limited. It is important to assess how the organization’s ethical climate perception and trust affect the decision of the whistleblowers. This study examines the impact of ethical climate and organizational trust on the intent of whistleblowing in hospitals.

## METHODS

The population of the study was composed of nurses and secretaries working in a university hospital with 800-beds in Ankara, Turkey. The study was conducted for three months in 2016. It included 368 nurses, 315 secretaries in the hospital. At the end of the study; a total of 369 questionnaires were obtained; 167 from nurses and 202 from secretaries. Consequently, using convenience sampling 54% of the participants replied to the questionnaire.

In order to determine the opinions of the nurses and secretaries about the ethical climate of the hospital Ethical Climate Questionnaire, which was developed by Victor and Cullen (1988)^6^ and revised by Cullen, Victor & Bronson (1993)^7^, was used. The scale consists 36 items and three dimensions and cover egoism, benevolence, and principled ethical climates. We found the reliability coefficient of the scale as *α*=0.876 and it was highly reliable.

In order to determine the participants’ level of organizational trust “The Organizational Trust Inventory Short Form” which was developed by Cummings and Bromiley (1996)^8^ was used. Cummings and Bromiley (1996)^8^ found that the validity of the short form of the scale was higher than the long form. For this reason, the short form of the scale was preferred in the study. The scale consists 12 items and two dimensions (cognitive and emotional trust). We found the reliability coefficient of the scale as α=0.930 and it was highly reliable.

In order to determine the participants’ intention of whistleblowing “Whistleblowing Scale” which was developed by Celep and Konakli (2012)^9^ was used. The scale consists 16 items and four dimensions (internal, external, anonymous and supporter whistleblowing). We found the reliability coefficient of the scale as *α*=0.921 and it was highly reliable.

In the study, structural equality models were examined by path analysis, and the models found meaningful were interpreted. For path analysis, Lisrel 9.2 (Student) version was used. Multi-regression analysis was also applied. To assess the socio-demographic features of the participants, descriptive statistics were used.

## RESULTS

### Socio-demographic characteristic of participants

About 91.1% of the participants were women, 65% were married and 62.6% had license degree. In addition, most of the participants were secretary (54.7%) and between the ages of 30 and 39 (54.7%) (Table-I).

**Table-I:** Socio-demographic characteristic of participants.

Variables	n	%
*Age*		
22-29	50	13.6
30-39	202	54.7
40-49	94	25.5
>50	23	6.2
*Gender*		
Male	33	8.9
Female	336	91.1
*Marital Status*		
Married	240	65.0
Single	129	35.0
*Education level*		
High school and associate degree	70	19.0
License degree	231	62.6
Master degree	68	18.4
*Position*		
Nurse	167	45.3
Secretary	202	54.7
*Length of employment*		
0-5 years	54	14.6
6-10 years	110	29.8
11-15 years	96	26.0
16-20 years	67	18.2
20 years +	42	11.4
*Total*	369	100.0

### Results of Regression Analysis

In the regression model, whistleblowing intention was dependent variable. The independent variables were dimensions of ethical climate (egoism, benevolence, principled), dimensions of organizational trust (cognitive and emotional), age, duration of profession, duration of work in the hospital, profession, gender, marital status, level of education and work place. All variables were included in the regression analysis with the forward selection method. Accordingly, dimensions of ethical climate, profession, and gender had a significant effect on overall whistleblowing intention. The obtained model was statistically significant at 95% confidence level (*p*<0.001). An increase in the egoist ethical climate by one point increases the whistleblowing intention by 0.572 points. An increase in the benevolence ethical climate by one point increases the whistleblowing intention by 0.377 points. An increase in the principled ethical climate by one point decreases the whistleblowing intention by 0.226 points. Mean scores of whistleblowing scores of secretaries were 0.161 points lower than nurses. It was also seen that the mean scores of whistleblowing intentions of females were 0.475 points lower than males. If work place is clinic whistleblowing intention score decreases 0.256 points compared to policlinic. 54.2% of the whistleblowing intention score was explained by the independent variables in the model (Table-II).

**Table-II:** The Effects of Ethical Climate and Organizational Trust Dimensions and Socio-demographic Characteristics on the Whistleblowing Intention.

Independent Variables	Dependent Variable (Whistleblowing intention)						

	Β	SE	t	p	Std. (B)	%95 Confidence Interval	

						Lower bound	Upper bound
Constant	1.052	0.313	3.358	0.001		0.436	1.668
Egoism	0.572	0.106	5.399	<0.001	0.250	0.364	0.781
Benevolence	0.377	0.042	9.023	<0.001	0.448	0.295	0.459
Principled	-0.226	0.071	-3.204	0.001	-0.154	-0.365	-0.087
Profession (Secretary)	-0.161	0.069	-2.345	0.020	-0.137	-0.296	-0.026
Gender (Female)	-0.475	0.077	-6.203	<0.001	-0.231	-0.626	-0.324
Work place (Clinic)	0.256	0.068	3.786	<0.001	0.219	0.123	0.390

R= 0.736; R2 = 0.542; F= 71.310; p<0.001

### PATH Analysis Models

Path analysis is defined as a method used to estimate the structural relationship between variables and to determine how much of the total effects of independent variables on dependent variables are directly and indirectly.^10^ The fit indices used in the study are shown in Table-III.

**Table-III:** Fit Indices of the Model.

Fit Indices	Good Fit*	Acceptable Fit*	Fit Status
Χ2/df	0 ≤^2^/df ≤ 2	2 ≤^2^/df ≤ 3	Acceptable fit
RMSEA	0 ≤ RMSEA ≤ 0.05	0.05 ≤ RMSEA ≤ 0.08	Good fit
CFI	0.97 ≤ CFI ≤ 1	0.95 ≤ CFI ≤ 0.97	Good fit
GFI	0.95 ≤ GFI ≤ 1	0.90 ≤ GFI ≤ 0.95	Good fit

**Fig.1:**
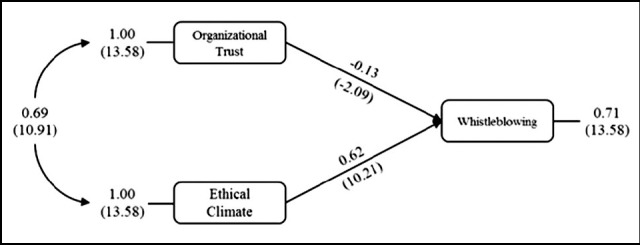
Effect of Organizational Trust and Ethical Climate on Whistleblowing.

In the first model, the situation was examined in which the intention of whistleblowing was dependent and the ethical climate and organizational trust were independent variables. Accordingly, in explaining the whistleblowing intention, the organizational trust score had negative effect; while the ethical climate score had the positive effect (Fig.1). The obtained model is statistically significant (X^2^=0.00; df=0; p=1; RMSEA=0.000<0.05; CFI=1>0.95; GFI=1>0.90).

When the model included both ethical climate and organizational trust as independent variables, the direct effect of ethical climate on the whistleblowing intention was 62%; the direct impact of organizational trust on the whistleblowing intention was 13% (Fig.1).

The values shown in parentheses in Fig.1 indicated the t values of the path coefficients and the path coefficients were statistically significant (*t*≥1.96; *p*≤0.05). In this model, there was a relationship between organizational trust and ethical climate, which was not causal and was represented by a double-headed curve. The direct effects of organizational trust and ethical climate on the whistleblowing intention could be seen but does not have indirect effects in the model.

**Fig.2:**
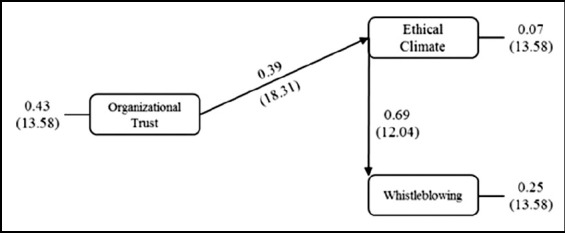
Effect of Organizational Trust on Whistleblowing When Ethical Climate is a Moderator.

In the second model, the situation was examined in which the organizational trust was an independent variable, ethical climate was a moderator, and whistleblowing intention was a dependent variable (Fig.2). The values shown in parentheses in Fig.2 indicated the t values of the path coefficients and the path coefficients were statistically significant (*t*≥1.96; *p*≤0.05). The obtained model was statistically significant X^2^=0.00; df=1; p=1; RMSEA=0.000<0.05; CFI=1.00>0.95; GFI=1.00>0.90).

Organizational trust had a direct effect of 39% on ethical climate. The ethical climate had a direct impact on the whistleblowing with a value of 69%. Organizational trust indirectly had a 27% (0.39*0.69) impact on the whistleblowing.

## DISCUSSION

It is possible to increase the quality and to provide sustainability of service provision by preventing the spread of negative behaviours and actions through whistleblowing. The fact that health care has vital qualities increases the importance of whistleblowing. In the health sector, negative events or behaviours that are subject to whistleblowing can cause results that are more concrete, serious and effect human lives. Therefore, it is important to determine the factors that affect the whistleblowing and to encourage it in the hospitals. Hospitals must first establish rules and ethical codes that will reduce unethical practice that would harm the patient, and encourage hospital employees to comply with these rules and regulations.

It was seen that the ethical climate score had a positive high level effect on whistleblowing intention. Thus, ethical climate is one of the most effective tools that can be used to influence ethical attitudes and behaviours of individuals. Elci, Erdilek Karabay & Akbas (2016)^11^ found that ethical climate influenced both internal and external notices in their study on public sector employees. Aslan & Akarcay Ulutas (2015)^12^ found that ethical climate and culture “error-reporting confidentiality” influenced negatively in the study they conducted on workers in public hospitals. In other studies^13^,^14^ conducted in different areas it was found that employees in organizations with more ethical climates had more whistleblowing intention.

In order to solve the problems related to whistleblowing, it is important to provide a strong ethical climate in the organization. It must include commitment to ethical behaviour starting from senior management and mandatory ethics training for all employees. Managers must provide an environment in which employees will demonstrate ethical behaviour. In environments where inappropriate behaviour is prevalent, managers can influence employee behaviour by changing the ethical climate. Organizations can promote ethical behaviour by identifying unethical behaviours, controlling and correcting these behaviours, creating ethical rules and policies, and putting them into action.

Health care workers in particular nurses have a dilemma about whistleblowing unethical incidents as it is perceived as disloyalty rather than moral behaviour, the behaviour of the whistleblowing can be low. It is important to determine clearly, which behaviours are acceptable in the organization. Ethical guidelines should be established which provides health workers information on ethical behaviour principles that they should obey when performing their duties when they are deciding or acting and to avoid the problem of how to use the authorities and resources in a fair, impartial, honest and consistent manner.

It was seen that the organizational trust score had a low level and negative effect on whistleblowing intention. Binikos (2008)^15^ suggested that the impact of organizational trust on whistleblowing may be contradictory. Low-level trust may cause not to be reported, high-level trust may also produce the same effect. Because employees will think that such bad behaviours will not be allowed to happen in their organizations without their need to report them and they will observe inappropriate behaviour.

The direct effect of organizational trust on whistleblowing intention was negative and low. Whereas in the case of ethical climate had a moderator role, the indirect effect of organizational trust on whistleblowing intention turned into positive and slightly increased. Therefore, the moderator role of ethical climate between organizational trust and whistleblowing intention is important. If the nurses and secretaries, who trust the hospital they work, perceive the ethical climate as high, their whistleblowing intention increases.

For health workers to report errors on a regular basis, they need to have a high degree of trust in their organization that they will not see an unfair response as a result of reporting.^16^ Arifah et al. (2017)^17^ found that the organizational trust has positive impact on whistle-blowing intentions. Employees who believe that the organization will support them in case they report a problem believe that something about the subject will change that a situation against him will not develop.^18^ Cakinberk, Dede & Yilmaz (2014)^19^ found that there is a negative relationship between organizational trust perceptions and organizational silence behaviours of employees. Seifert et al. (2014)^20^ examined the relationship between trust, organizational justice and whistleblowing, and found that participants with a certain level of trust tend to report more and when they have trust in both their managers and their organizations, they have the highest level of whistleblowing intention. According to Cemaloglu (2017)^21^, the reaction of the management of the organization to the unethical and illegal events in the past has an effect on the whistleblowing behaviour.

Employees want to be confident that they will be treated with sensitivity and fairness and that they will not face any retaliation in case of informing the management of the wrong practices they are facing in the organization. To provide a cultural exchange that will create an atmosphere of trust in health care all factors that can provide trust such as openness, autonomy, shared values, benevolence, honesty, communication, empowerment, organizational culture and ethical climate should be taken into consideration. If employees trust the management and believe that the organization will protect themselves and they will not lose their personal rights, they will resort to whistleblowing behaviour for all unethical and illegal activities.

It is important that nurses and secretaries, who have a significant role in the provision of the health care service, to report negative events or situations when they encounter to reduce unethical practice. It is suggested that steps should be taken to increase the ethical climate perceptions and organizational trust levels of nurses and secretaries.

### Author’s Contribution

**SA** **&** **SK:** Designed the study.

**SK:** Collected the data.

All authors prepared the manuscript and approved the final version to be published.
